# The German Version of the Strengths Use Scale: The Relation of Using Individual Strengths and Well-being

**DOI:** 10.3389/fpsyg.2017.00637

**Published:** 2017-04-27

**Authors:** Alexandra Huber, Dave Webb, Stefan Höfer

**Affiliations:** ^1^Department of Medical Psychology, Medical University of InnsbruckInnsbruck, Austria; ^2^Institute of Psychology, University of InnsbruckInnsbruck, Austria; ^3^Marketing (UWA Business School), University of Western Australia (M263), CrawleyWA, Australia; ^4^Institute of Psychology, The Health and Life Sciences UniversityHall in Tirol, Austria

**Keywords:** character strengths, Strengths Use Scale, positive psychology, well-being, German validation

## Abstract

Theoretical perspectives in positive psychology have considered the possession and use of strengths equally but in applied research more studies focused on having them, probably due to the absence of psychometrically adequate scales. Therefore, the aim of this study was to assess the psychometric characteristics of the German language version of the Strengths Use Scale (SUS) and to explore relationships between strengths use and several indicator measures of well-being: the presence of positive and the absence of negative affect, self-esteem as identity aspect, vitality as self-regulatory resource, and stress for capturing the evaluation of difficulties and obstacles impinging on well-being. The original English version of the SUS was translated following recommended independent forward-backward translation techniques. Exploratory and confirmatory factor analyses were conducted, including a German-speaking convenience sample of university students (*N* = 374). Additionally, the relations of strengths use and well-being indicators were analyzed. Factorial validity revealed a single-factor structure of the German version of the SUS, explaining 58.4% variance (factor loadings: 0.58 to 0.86), approving the scale’s design and showing high internal consistency (Cronbach’s α 0.95). The hypothesized positive relationships of strengths use with positive affect, self-esteem, and vitality were confirmed as well as the negative relationships with negative affect and stress. The German version of the SUS is psychometrically sound and data indicate that individual strengths use and well-being related measures interact. The instrument can be recommended for future research questions such as if and how the promotion of applying individual strengths during education enhances levels of well-being, or how the implementation of strengths use in job-design guidelines or working conditions can result in higher levels of well-being or healthiness.

## Introduction

Positive psychology deals with the optimal functioning of humans, or rather, with factors that make life most worth living (Seligman and Csikszentmihalyi, 2000). Its aim is to enrich the science of psychology by focusing on positive aspects of human experience and behavior (Seligman and Csikszentmihalyi, 2000; Peterson and Seligman, 2004) comprising the research objective to explore what contributes toward the maintenance of mental health, happiness, and well-being. Research on well-being can be distinguished into two traditions (Deci and Ryan, 2008). In one – the hedonistic tradition – the focus is on happiness, generally defined by life satisfaction, the presence of positive and the absence of negative affect (subjective well-being; Diener, 1984). In the other – the eudaimonic tradition – the focus is on living life in a deeply satisfying way including engagement with existential challenges of daily life (psychological well-being) concerning, e.g., autonomy, self-acceptance, personal growth, positive relations with others, environmental mastery, and the experience of purpose in life (Ryff and Keyes, 1995). Historically, in favor of studying psychopathology, the science of psychology primarily focused on negative aspects of human experiences, e.g., mental disorders, their diagnosis and treatment (Cassell, 2002; Sheldon and Lyubomirsky, 2004; Harzer and Ruch, 2013). But today’s amount of literature on positive psychology and well-being reflects a paradigm shift, confirming the importance of describing, measuring, and implementing factors that maintain and promote mental health in respect to happiness, engagement, and self-actualization (Kahneman et al., 1999; Fredrickson, 2011).

A key aspect of positive psychology has been the focus on character strengths, which arguably play a crucial role in human functioning and flourishing (Seligman, 2002a,b). One example is the VIA-classification describing 24 character strengths assigned to six different virtues, which have been theoretically considered as being important for over 3000 years across many religions, cultures, and traditions (Peterson and Seligman, 2004). The VIA-IS (Peterson and Seligman, 2001; Peterson and Park, 2009) is one among several instruments to assess these 24 character strengths. They are conceptualized as positive, stable, and moral traits or characteristics. Seligman (2002a) suggests that rather than focusing on the correction of weaknesses to obtain well-being and to maximize their positive benefits, individuals ought to utilize their character strengths by expanding and bringing them to bear according to life circumstances.

Other concepts of strengths focus on, e.g., occupational settings (Buckingham and Clifton, 2001) or a more general application of strengths in work and life (Linley and Harrington, 2006). These concepts define strengths as natural capacities coming from within that we yearn to use, that enable authentic expression and energize us (Govindji and Linley, 2007), and which belong to positive traits and/or psychological capacities/talents refined with knowledge and skills (Clifton and Anderson, 2002; Proctor et al., 2011). Therefore, strengths ‘reflect a pre-existing capacity for a particular way of behaving, thinking, or feeling that is authentic and invigorating, and can enable optimal functioning, development and performance in the pursuit of valued outcomes’ (Linley and Harrington, 2006; Linley, 2008). Strengths are supposed to be largely stable but can be developed more or less by psychological activities and experiences. Like personality can adapt to situational demands (but being consistent over time; Fleeson, 2001) strengths also may fluctuate according to different situations, but will remain largely stable. This definition represents a broader approach as it does not imply an assumed moral valence that goes beyond the positive valence associated with the term ‘strength’ (cf. Peterson and Seligman, 2004). In this sense strengths may include moral virtues or character strengths, but are not limited to them.

Theoretical perspectives in strengths research have focused equally on possessing/knowing and using/applying them. Despite the preliminary evidence, indicating that strengths use, rather than having or knowing one’s strengths, contributes to well-being (Govindji and Linley, 2007; Rath, 2007), prior studies have tended to initially concentrate more on having low or high levels of strengths than on applying them (Park et al., 2004; Peterson et al., 2007; Gander et al., 2013). Moreover, research on character strengths as conceptualized by the VIA-classification including inter-relationships with various situational, personal, and environmental variables is expanding, whereas positive psychological research examining more generic aspects of strengths (as personal characteristics and/or individual natural talents refined with knowledge and skills) has been somewhat disregarded.

Character strengths result in more positive emotions, engagement, meaning, accomplishment, and better relationships (Seligman, 2011) and contribute to subjective well-being, mental and physical health and life satisfaction (Peterson and Seligman, 2004; Peterson and Park, 2011; Douglass and Duffy, 2014). Hope, zest, gratitude, curiosity, and love were identified as being the most strongly related character strengths to life satisfaction, whereas modesty, creativity, open-mindedness, appreciation of beauty, and love of learning were least related (Park et al., 2004). In a Swiss study for example, the character strengths of hope, zest, love, social intelligence, and perseverance were most strongly associated with life satisfaction, and hope, zest and humor were consistently the highest correlated character strengths with well-being (MartínezMartí and Ruch, 2014). These results show an overall positive link between character strengths and well-being, with some being apparently more influential. Even higher levels of subjective and psychological well-being and health can be achieved by applying character strengths (e.g., Lyubomirsky et al., 2005; Seligman et al., 2005; Gander et al., 2013; Proyer et al., 2014). For example, character strengths use (hope, zest) has been shown to predict subjective well-being and correlated positively with self-esteem in a sample of UK students (Proctor et al., 2011) as well as with positive affect for a sample of UK students (Ouweneel et al., 2014). Higher levels of well-being were also found in studies focusing on the application of specific character strengths like, e.g., gratitude (Emmons and McCullough, 2003), kindness (Otake et al., 2006) or humor (Gander et al., 2013). The effect of character strengths-based interventions (e.g., using your strengths in a new way every day for 1 week; Seligman et al., 2005) on increasing well-being and decreasing depression over time has also been documented (Gander et al., 2013; Proyer et al., 2014).

Recent research into strengths use has shown that, similar to specific VIA-character strengths, general strengths use is positively associated with well-being, also over time (Wood et al., 2011). In a study with UK undergraduate university students, findings revealed that individuals who use their strengths experience greater subjective well-being, and that increased subjective well-being is related to both mental and physical health-related quality of life (Proctor et al., 2011). Higher levels of happiness, fulfillment and well-being in terms of subjective (affective balance, satisfaction with life) and psychological (engagement with existential life challenges) well-being can also be accomplished by strengths use (Govindji and Linley, 2007), whereas possession/knowledge was no significant independent predictor of either, suggesting that it is more important to use your strengths rather than simply to know what they are. Strengths use correlated positively with vitality for a sample of French-speaking Canadian workers (Dubreuil et al., 2014), with positive affect for a sample of US students (Douglass and Duffy, 2014) and increased resilience was observed when using strengths like, e.g., ability to relax, amusement, or optimistic thinking (Fredrickson et al., 2003). Further results in this research area showed that strengths also may function as buffers against negative effects like stress or the development of psychological problems. For example, one study including teenagers found that greater numbers of personality strengths (such as sociability, optimism, self-confidence, empathy, expressiveness, faith, internal locus of control, social orientation to problem solving) promoted resilience, good functioning in academic/social domains and mental health in regard to not develop psychiatric disorders (Bromley et al., 2006). In summary, these findings indicate that strengths and strengths use are related to increased well-being and life satisfaction, and that strengths may function as a buffer against negative life outcomes.

Different instruments measuring the application of (character) strengths have been developed. The ‘Applicability of Character Strengths Rating Scales’ aims to measure the extent to which each of the 24 character strengths of the VIA-IS is applicable in work and private life (Harzer and Ruch, 2013), whereas the ‘Strengths Use Scale’ (SUS; Govindji and Linley, 2007), being the core of this study, was developed for a more generic use. Following their definition of ‘strengths,’ people can interpret for themselves the meaning of their strengths including personal, physical, and psychological strengths as well as character strengths (e.g., sportiness, intelligence, manual skills, financial intuition, cooking, health maintaining strategies, multi-cultural competence, peer resistance, perfectionism, organizational abilities,…).

Wood et al. (2011) also raised the question why research has preliminary more tended to focus on the effects of low versus high strengths possession and in particular why several perspectives have focused exclusively on character strengths (e.g., Peterson and Seligman, 2004). The authors suggested that this may have been due to the absence of psychometrically adequate scales to measure general strengths use in the past. In making this suggestion the authors nonetheless acknowledge the original work of Govindji and Linley (2007), who developed the SUS to assess the use of all individual kinds of strengths in a general adult population, providing a preliminary empirical basis and giving implications for strengths coaching and counseling or coaching psychologists. Following principal components analysis, the original authors revealed from an initial pool of 19 items, the existence of a single ‘strengths use factor’ comprising of 14 items with factor loadings of between 0.51 and 0.79. In spite of high reported reliability (Cronbach α 0.96), no information is provided concerning the convergent validity of the scale suggesting the need for further empirical testing. In response, Wood et al. (2011) followed a more comprehensive testing of the scale to find support for a single-factor structure as suggested by Govindji and Linley (2007).

The original English version of the SUS has already been translated into Hebrew (Littman-Ovadia et al., 2014) with good reliability (Cronbach α from 0.88 to 0.92) in a study focusing on strengths-based career counseling. Moreover, a work-adapted SUS version built upon the original scale is existing in English (Kong and Ho, 2015) and French (Dubreuil et al., 2014) as well as a Dutch translation (van Woerkom and Meyers, 2015), partially built upon the ‘Strengths Knowledge Scale’ and the SUS (Govindji and Linley, 2007). Previous studies using the SUS (e.g., Proctor et al., 2011; Wood et al., 2011; Douglass and Duffy, 2014, 2015; Quinlan et al., 2015; Rickard et al., 2016) already contributed to the continued international interest to extend the exploration of generic strengths application in other cultural and geographic contexts (Norrish and Vella-Brodrick, 2009).

Therefore, this study investigates the psychometric properties of the German version of the SUS using exploratory and confirmatory techniques. Consistent with Wood et al. (2011), this study further explores the relationships between strengths use and several indicator measures of well-being. The compilation of momentary negative and positive affect for measuring an emotional component of well-being (Watson et al., 1988), stress for capturing the evaluation of difficulties and obstacles impinging on well-being (Cohen and Williamson, 1988), self-esteem for examining the self/identity aspect of well-being (Rosenberg, 1965) and vitality for analyzing the availability of sufficient self-regulatory resources to successfully navigate the challenges of daily life (Ryan and Frederick, 1997) was used, based on the indicator measures proposed by Wood et al. (2011). Therefore, positive relationships with positive affect, self-esteem, and vitality and negative relationships with negative affect and stress are hypothesized. Consequently, in combination, the present analyses build on the work of Govindji and Linley (2007) as well as Wood et al. (2011) in a German-speaking context.

## Materials and Methods

### Translation

The translation of the questionnaire from English to German followed recommended forward and backward translation techniques (Marquis et al., 2005). To commence, two independent German native speakers with excellent English language skills translated the original version into the German language. Thereafter, one homogeneous version was developed by an iterative discussion process in consensus meetings. Two English native speakers with excellent German language skills independently back-translated the scale. Again, one consistent version of both back-translations was drafted and the original author of the scale was consulted about the final version. As there were no concerns raised at either stage, the German version of the SUS was considered an accurate linguistic reflection of the original survey and, was subsequently administered to participants to commence the psychometric evaluation process.

### Sample and Procedure

After institutional review board approval was given, university students received an electronic invitation via email as one optional possibility to fulfill the work assignment within a student course. They were asked to complete the German version of the SUS, the Perceived Stress Scale, the Positive and Negative Affect Schedule, the Self-Esteem Scale and the Subjective Vitality Scale online. Furthermore a subsample of students was encouraged to recruit additional participants. Altogether 374 people participated (females 67.9%; mean age 28.0 ± 11.4 ranging from 18 to 85); for further details please see **Table 1** (data missing if sample sizes do not equal N or 100% for each group).

**Table 1:** Socio-demographic characteristics.

		*N*	%
Total cohort		374	100
Gender	Female	250	66.8
	Male	118	31.6
Age	(*M* ±*SD*)	28.0 ± 11.4	
Partnership	Yes	195	52.1
	No	173	46.3
Children	Yes	49	13.1
	No	319	85.3
Education	Compulsory school	2	0.5
	Vocational training	25	6.7
	University qualification	208	55.6
	University degree	134	35.8
Satisfaction with	Yes	284	75.9
current health status	No	84	22.5

*M, mean; N, number of patients; SD, standard deviation.*

### Measures

#### Strengths Use Scale

The German language translation (Fragebogen zur Anwendung von Stärken; FAS) of the English original version (Govindji and Linley, 2007) was used to measure strengths use, giving the original instruction: ‘The following 14 questions ask you about your strengths, that is, the things that you are able to do well or do best. Respond using a 1 (strongly disagree) to 7 (strongly agree) scale.’ The scale consisting of 14 items (e.g., ‘I am able to use my strengths in lots of different ways’ or ‘Using my strengths comes naturally to me’) originally reported an excellent internal consistency and has been replicated in this sample with Cronbach’s α 0.95 (a copy of the German language version can be found in the electronic Supplementary Material).

#### Perceived Stress Scale

The German version of the Perceived Stress Scale (Büssing, 2011) with 10 items (five-point format from 0 = ‘never’ to 4 = ‘very often’) was used to measure the extent to which respondents experienced unpredictable, uncontrollable, and overloaded situations in their lives during the prior month (e.g., ‘How often have you felt that you were unable to control the important things in your life?’ or ‘How often have you felt that you were on top of things?’). Cronbach’s α was 0.84 in the present sample.

#### Positive and Negative Affect Schedule

Positive and negative affect were operationalized with the German version (state instruction) of the Positive and Negative Affect Schedule (Krohne et al., 1996). The scale consisting of 20 items, 10 describing positive (e.g., excited, inspired, and attentive) and 10 negative emotional conditions (e.g., hostile, irritable, and afraid), was assessed using a five-point 1 = ‘very slightly’ or ‘not at all’ to 5 = ‘extremely’ scale format. Internal consistencies were Cronbach’s α 0.82 for positive affect and 0.87 for negative affect in the present sample.

#### Self-esteem-scale

The German version of the Rosenberg Self-Esteem Scale (Ferring and Filipp, 1996) was adopted to evaluate the global self-esteem of participants. Positive and negative feelings about the self were measured with 10 statements (e.g., ‘I feel that I have a number of good qualities’ or ‘I wish I could have more respect for myself’) using a four-point 1 = ‘strongly disagree’ to 4 = ‘strongly agree’ scale format. Internal consistency of Cronbach’s α 0.90 was found in the present sample.

#### Subjective Vitality Scale

Vitality was measured with the German version of the Subjective Vitality Scale (Ryan and Frederick, 1997). The scale, which adopts a 1 = ‘not true at all’ to 7 = ‘very true’ format, comprises of six items which are considered to reflect aspects of psychological well-being and indicate one’s level of vitality and, the subjective feeling of being alive and alert (e.g., ‘I feel alive and vital’ or ‘I have energy and spirit’). Cronbach’s α was 0.88 in the present sample.

### Statistical Analyses

For all statistical analyses IBM SPSS Statistics 22 (IBM Corp, 2013) and AMOS (Arbuckle, 2013) was used. Means ± standard deviation (*M* ±*SD*) and Pearson’s coefficient inter-correlations were calculated to describe metric properties of the items and the scales. The skew and kurtosis of the SUS shed light on the data distribution where Miles and Shevlin (2001) state, that total values smaller than 1 indicate no violation of normal distribution.

An EFA with a maximum likelihood method of estimation was conducted, and moreover a parallel analysis (Horn, 1965), to determine the numbers of factors to extract. Therefore, the Kaiser-Meyer-Olkin-measure for sampling adequacy should range from 0 to 1, with a value of 0.50 being suitable for factor analysis and Bartlett’s test of sphericity should be significant (*p* < 0.05) (Williams et al., 2010). Squared factor loadings, indicating the amount of variance in each variable that is accounted for, should exceed >0.50 within a sample size of 300 to 500 participants for being considered as good (MacCallum et al., 1999). A CFA was conducted to determine if the hypothesized structure of the German SUS fits the data well. Using the maximum likelihood method of estimation, the χ^2^, df, TLI, CFI, and RMSEA with lower and higher bounds of the 90% confidence interval (LO90; HI90) are reported as measures of global fit. χ^2^ is recommended not to be significant (p) when 100 < *N* < 300 (Hair et al., 2010) as this fit index is dependent on the number of subjects. Values not smaller than 0.95 for CFI and values not larger than 0.08 for RMSEA are considered as indicators of good global fit (Hu and Bentler, 1999; Schermelleh-Engel et al., 2003). In case of insufficient initial model fit, modification indices were considered to improve the model after ensuring theoretical justification.

Reliability was determined by using composite reliability as an amendment to Cronbach’s α, which often understates reliability. Cronbach’s α indicates acceptable internal consistency when values are >0.70 (Nunnally, 1978; see Peterson, 1994) and composite reliability values ≥0.70 can be considered as being good values (Hair et al., 2010). Additionally to asses test-stability test-retest data was collected over a 2 days interval (*N* = 54; females 75.9%; mean age 23.6 ± 5.3 ranging from 20 to 56; 53.7% with partnership, 85.2% with university qualification). A comprehensive measure of convergent validity (average variance extracted; Fornell and Larcker, 1981) was obtained with:

$$\frac{\Sigma {\text{ of standardized loading}}^{2}}{\Sigma {\text{ of standardized loading}}^{2}\;+\;\Sigma \text{ of error terms}}$$

It measures the amount of variance that is captured by the construct specifically in relation to the amount of variance due to measurement error. Convergent validity is established if the shared variance accounts for 0.50 or more of the total variance (Fornell and Larcker, 1981).

## Results

Descriptive statistics for all study variables including *M* ±*SD*, Cronbach’s α, minimum/maximum scores and observed ranges are presented in **Table 2**, confirming the internal consistency requirement of >0.70 within all instruments. Pearson’s coefficient inter-correlations between the study variables are presented in **Table 3**, showing constant highly significant correlations (*p* < 0.001), except between positive and negative affect. The total skew value showed a negative skewness of -0.78 and the kurtosis a positive value of 0.12, meeting the recommendation of values being smaller than 1. Test–retest stability was 0.88.

**Table 2:** Descriptive statistics for all study variables.

	*M* ±*SD*	α	Min. | Max.	Observed range
Negative affect	1.72 ± 0.67	0.87	1.0 | 5.0	1.0–4.0
Positive affect	3.21 ± 0.68	0.82	1.0 | 5.0	1.3–4.7
Self-esteem	3.78 ± 0.78	0.90	1.0 | 5.0	1.5–5.0
Strengths use	4.95 ± 1.14	0.95	1.0 | 7.0	1.4–7.0
Stress	1.72 ± 0.68	0.84	0.0 | 4.0	0.0–3.4
Vitality	4.81 ± 1.20	0.88	1.0 | 7.0	1.2–7.0

*α, Cronbach’s alpha; M, mean; Max., maximum score; Min., minimum score; SD, standard deviation.*

**Table 3:** Pearson’s coefficient inter-correlations between strengths use and indicator measures.

	Strengths use	Negative affect	Positive affect	Self-esteem	Stress	Vitality
Strengths use	-					
Negative affect	-0.43***	-				
Positive affect	0.43***	-0.13*	-			
Self-esteem	0.64***	-0.52***	0.47***	-		
Stress	-0.57***	0.46***	-0.40***	-0.58***	-	
Vitality	0.69***	-0.49***	0.46***	0.60***	-0.61***	-

***^∗^***p* < 0.05, **^∗∗∗^***p* < 0.001. All two-tailed tests.*

The sample was found to be suitable for EFA (Kaiser-Meyer-Olkin-measure: 0.959; Bartlett’s test of sphericity: *p* < 0.001). Employing a maximum likelihood method of estimation and further a parallel analysis (Horn, 1965), a single-factor solution explaining 58.4% variance with factor loadings ranging between 0.58 and 0.86 resulted (**Table 4**). The first factor had an eigenvalue of 8.60, with the remaining values clearly below the point of intersection (0.855 to 0.172). The squared factor loadings were mostly satisfying (>0.50) except item no. 2, 7, and 12 (0.336; 0.384; 0.410).

**Table 4:** Means ± standard deviations, skew, kurtosis and factor loadings of the German SUS.

Items (1 ‘strongly disagree’ → 7 ‘strongly agree’)	*M* ±*SD*	Skew | Kurtosis	Factor loadings
(1) I am regularly able to do what I do best.	5.07 ± 1.35	-0.791 | 0.382	0.70
(2) I always play to my strengths.	4.44 ± 1.37	-0.235 |-0.324	0.58
(3) I always try to use my strengths.	5.38 ± 1.39	-0.905 | 0.478	0.79
(4) I achieve what I want by using my strengths.	5.06 ± 1.40	-0.669 | 0.041	0.73
(5) I use my strengths every day.	4.58 ± 1.50	-0.422 |-0.379	0.77
(6) I use my strengths to get what I want out of life.	5.05 ± 1.50	-0.650 |-0.153	0.77
(7) My work gives me lots of opportunities to use my strengths.	4.72 ± 1.61	-0.514 |-0.551	0.62
(8) My life presents me with lots of different ways to use my strengths.	5.16 ± 1.48	-0.885 | 0.321	0.77
(9) Using my strengths comes naturally to me.	5.33 ± 1.57	-0.847 |-0.038	0.85
(10) I find it easy to use my strengths in the things I do.	4.75 ± 1.54	-0.439 |-0.511	0.82
(11) I am able to use my strengths in lots of different situations.	4.94 ± 1.46	-0.691 | 0.064	0.86
(12) Most of my time is spent doing the things that I am good at doing.	4.77 ± 1.35	-0.598 | 0.042	0.64
(13) Using my strengths is something I am familiar with.	5.25 ± 1.51	-0.942 | 0.375	0.83
(14) I am able to use my strengths in lots of different ways.	5.11 ± 1.44	-0.753 | 0.024	0.86

*M, mean; SD, standard deviation; SUS, Strengths Use Scale.*

**FIGURE 1:**
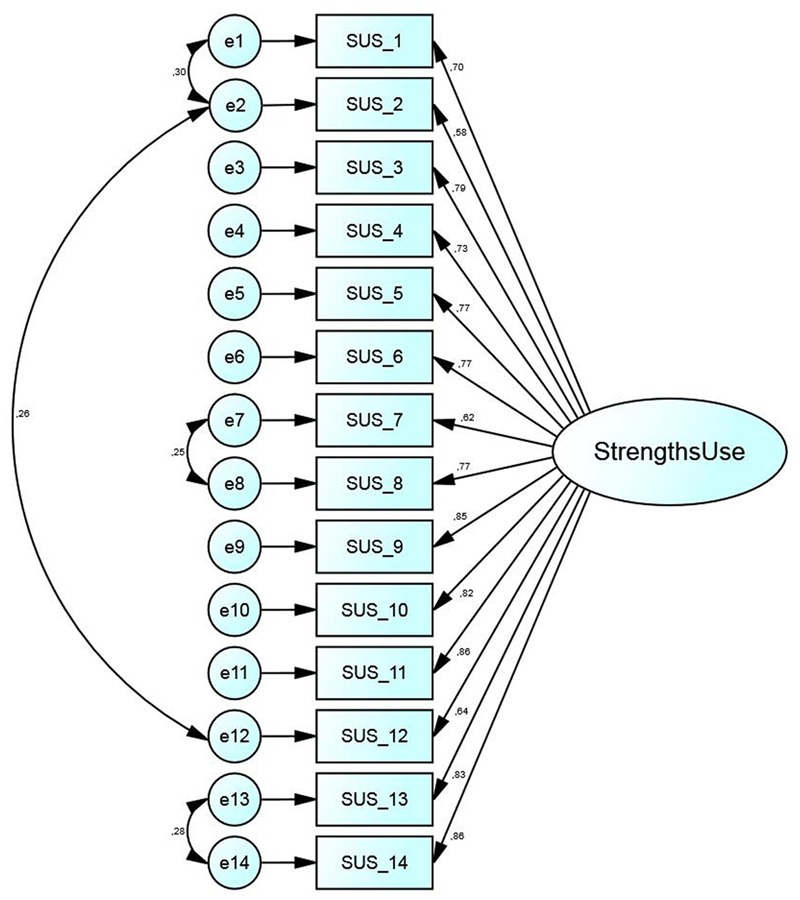
**Factorial structure of the German SUS**.

The initial model fit of the German version of the SUS was not sufficiently satisfying (χ^2^: 338.46; df: 77; *p* < 0.001; TLI: 0.92; CFI: 0.93; RMSEA: 0.096; LO90: 0.085; HI90: 0.106). CFA modification indices revealed that improvements to the SUS structure could be achieved by allowing four correlating error terms within the construct, leading to a satisfactory fit (χ^2^: 228.37; df: 73; *p* < 0.001; TLI: 0.95; CFI: 0.96; RMSEA: 0.076; LO90: 0.065; HI90: 0.087). The error terms (e1↔e2: 0.30; e2↔e12: 0.26; e7↔e8: 0.25; e13↔e14: 0.28) can be explained by similar item wording and content. Therefore, the error terms were allowed to covary as all items load on the same ‘strengths use’ factor sufficiently and are not logically causally, but merely statistically correlated (Brown, 2015). Cronbach’s α as well as composite reliability was 0.95 within this analysis, indicating excellent reliability. Furthermore an acceptable average variance extracted of 0.58 was achieved, meeting the requirement of convergent validity. Based on the presented results, the 14-item final German version of the SUS appears to reflect a valid and reliable version of the original English instrument (**Figure 1**) (Govindji and Linley, 2007).

As hypothesized, positive inter-correlations were found between strengths use and positive affect (*r* = 0.43), self-esteem (*r* = 0.64) and vitality (*r* = 0.69), whereas negative inter-correlations were found between strengths use and perceived stress (*r* = -0.57) and negative effect (*r* = -0.43). Therefore, testing relationships between strengths use and indicator measures of well-being, results showed a highly significant impact on all different facets, in particular concerning self-esteem and vitality.

## Discussion

In this study, exploratory and confirmatory approaches were taken to validate the German version of the SUS. The analyses confirmed the uni-dimensionality of the scale measuring one single strengths use factor, consistent with prior research (Govindji and Linley, 2007; Wood et al., 2011). Demands on internal consistency, composite reliability and convergent validity were met. Furthermore, all well-being indicator measures (positive and negative affect, self-esteem, stress, vitality) correlated with strengths use as hypothesized. These findings suggest that the German version of the SUS is comparable to the original English version, representing a valid and reliable measure of strengths use in the German language.

All items of the German version of the SUS loaded sufficiently on a single strengths use factor. But according to MacCallum et al. (1999), where the factor loading of an item should be at least 0.70 to be reasonably kept in an instrument, item no. 2 (‘I always play to my strengths’; 0.58), no. 7 (‘My work gives me lots of opportunities to use my strengths’; 0.62) and no. 12 (‘Most of my time is spent doing the things that I am good at doing’; 0.64) could be considered for elimination. However, other authors recommend to keep items in an instrument when the scale meets convergent validity and their factors load >0.60 (Huber et al., 2007; Hulland, 1999) or at least >0.40 (Hair et al., 2010). The authors of the original English version of the SUS (Govindji and Linley, 2007) revealed by conducting a principal components analysis from an initial pool of 19 items three components with eigenvalues greater than one, with a scree test showing one component above a marked elbow. A further analysis of the 14 items comprising this component showed one single ‘strengths use’ factor with loadings all >0.50 (56.2% total variance). These 14 items were then taken forward to constitute the original SUS (Govindji and Linley, 2007). In the original version, item no. 12 shows the lowest factor loading (0.51), followed by item no. 6 (0.52) and then no. 7 (0.53), whereas item no. 2 has a slightly higher factor loading (0.67) than in the German version (0.58). Altogether, it seems that item no. 2 (in the German version), no. 7 and no. 12 (in both versions) explain less variance then the others, maybe according to different constructs behind (e.g., self-confidence that one has strengths, and consequently uses them; or self-actualization). Moreover, all three items below the factor loading criterion of 0.70 in the German version of the SUS were part of the correlating error terms, item no. 2 even twice (e1↔e2; e2↔e12; e7↔e8). This might be due to similar wording (no. 1, 2, 12), imprecise translation (no. 2) or an ‘environmental view’ if there are opportunities to use one’s strengths (no. 7, 8). No information was found on error terms in the original version. These findings suggest thinking on a possible improvement by reducing the number of items of the German SUS in a further step. However, the requirements of a single strengths use factor were met for all 14 items statistically and with regards to content (c.f. Hair et al., 2010).

The German version of the SUS used the same instruction as prior studies (Wood et al., 2011), namely: ‘The following 14 questions ask you about your strengths, that is, the things that you are able to do well or do best. Respond using a 1 (strongly disagree) to 7 (strongly agree) scale.’ This instruction relates on the one hand to strengths use, and to their valued outcomes on the other. But there is no explanation of what strengths are or how they are defined. So it is questionable if participants really referred to ‘natural capacities coming from within that we yearn to use, that enable authentic expression, energize us and belong to positive traits and/or psychological capacities/talents refined with knowledge and skills.’ They could have thought on character strengths like in the VIA-classification but also on any other ways of behaving, thinking, or feeling in various situations or amoral behaviors with valued outcomes (e.g., lying, stealing). Basically, this can be interpreted as an advantage of this generic approach, but when there is no given definition of what is understood by ‘strengths’ and their use, artifacts can possibly occur and people may refer to aspects such as self-efficacy, self-actualization, self-confidence, situational daily life experiences from private and/or work life, personality, linguistic differences, cultural backgrounds, and the like in their answers. Therefore, a definition in the introduction of the instrument could be adjuvant when examining strengths use more generically, and to further benefit from this approach, participants should be given the opportunity to state to what kind of strengths they are referring to (e.g., open text field, asking in an interview) when interested in the content, which has been never done before.

This study replicated hypothesized relationships between strengths use and several indicator measures of well-being. The relationship between strengths use and affect has been primarily investigated either as an aspect of subjective well-being (beside life satisfaction; Diener, 1984) or, as a calculated composite variable (see Proctor et al., 2011). As strengths use is a positively defined construct in the pursuit of valued outcomes, one can assume that people using their individual strengths will experience more inspiration, enthusiasm, excitement, and the like, leading to more positive and less negative affect, momentary and permanently. Subsequently, because of the invigorating effect of strengths use to the core, these people will also possess more global self-esteem in terms of an individual’s sense of self, personal and social identity, worth and acceptance, and vitality in terms of the subjective feeling of being alive and energetic. A possible factor impairing one’s well-being can be stress. In general, stress is a cognitive-affective state that occurs when an individual perceives that the demands of an external situation are beyond his or her perceived ability to cope (Lazarus, 1966). This type of stress can also be termed as ‘distress’ and is detrimental to health (Cohen and Williamson, 1988). Elevated levels of stress hormones like cortisol/adrenaline are fine in the short-term, but in the long-term they can affect the immune system and lead to greater susceptibility to illness. According to Lazarus and Folkman (1984) stress is not objective but is evaluated individually by primary (challenge, threat, and harm/loss), secondary (problem-, emotion-, or appraisal-oriented coping) and re-appraisal in various situations. Therefore, in this analysis stress could have been examined more carefully as many variables (e.g., duration, form and level of stress occurring in different situation) influence the relation with well-being, and consequently also strengths use.

Beyond this, applying one’s strengths seems to be particularly more important than simply knowing what they are (Govindji and Linley, 2007). This reinforces the theoretical notion that people are intrinsically motivated to use their individual strengths (Linley et al., 2010), and that when they do so, they experience authenticity, vitality, and well-being (Peterson and Seligman, 2004). The present results suggest that applying all kinds of personal, physical, and psychological strengths positively influences affect, stress, self-esteem, and vitality. As the burden of mental illness continues to grow (World Health Organization, 2016), increased awareness is rising, that using individual strengths can foster resilience to better cope with daily challenges and stress in order to enhance well-being.

## Limitations and Future Research Suggestions

The study reported here is limited to cross-sectional data gathered from a convenience sample with a subjective assessment only. To enable findings and implications to be generalized to the broader population, future studies would benefit from data gathered longitudinally external to a university setting, also including different languages, cultures or behavioral measures.

A further point to think about is the ignorance of strengths on which participants are referring to. The introduction of the SUS does not provide an example definition of strengths. They could be understood as character strengths, but also as talents, skills, behaviors, or any other ‘strengths related’ outcome. Therefore, when applying the SUS in the future it might be beneficial to give people an opportunity (open text field, asking in an interview) to describe what they mean by ‘their strengths’ when interested in the content. Moreover, the indicator variables of positive/negative affect, self-esteem, vitality and particularly stress were primarily chosen to replicate prior study findings (Wood et al., 2011). Positive and negative affect can be measured as state (capturing emotional responses at the very moment) or trait (capturing mood or personality differences in emotionality; Watson et al., 1988). In SWB research, usually longer time frames of affect are considered. However, in this study rather temporary moods and emotions were captured than a longer term affective evaluation. Self-esteem comprises the individual sense of the self, worth, acceptance, personal and social society, therefore being a sensitive measure of well-being (Hewitt, 2005), whereas vitality represents a more dynamic aspect of well-being, capturing the availability of sufficient self-regulatory resources to successfully manage daily life challenges (Ryan and Frederick, 1997). Depending on different definitions of well-being (e.g., Diener, 1984; Ryan and Deci, 2000; Su et al., 2014) the chosen indicator variables are included or not. Stress, capturing the evaluation of difficulties and obstacles, is related to well-being but not defined as part of it in any definition (momentary negative affect addresses the only somewhat similar construct). This needs to be considered when interpreting the data and addressed accordingly in future research.

The importance of contextual factors in positive psychology has been acknowledged already (Seligman and Csikszentmihalyi, 2000). Stokols (2003) has argued for an identification of environmental conditions that either constrain individuals from realizing their strengths or, alternatively, enhance the opportunity to apply them. Indeed, situations vary in their capacity to foster or constrain human agency (Mischel, 1968). While the study presented here explored the application of individual strengths in a new cultural context, it is limited in not considering the relevance of the situational environment in respect to the presented correlations. Accordingly, an opportunity for future studies is to explore not only the correlations presented herein, but also others yet to be identified across a range of contexts.

Other studies within this research field suggest the existence of an even more complex inter-correlational model including some of the indicator measures of well-being used in this study. The possibility of these constructs being mediators or moderators has been discussed in the literature. Moderators can be defined as qualitative or quantitative variables that affect the direction and/or strength of the relation between a predictor and a criterion variable. Mediators can be defined as variables accounting for the relation between a predictor and a criterion in a certain extent. Whereas moderator variables specify when certain effects will hold, mediators speak to how or why such effects occur (Baron and Kenny, 1986). For example, previous studies found positive affect to be a moderator of the relationship between gratitude and subjective well-being in school children and adolescents (Froh et al., 2009), self-esteem to be a mediator between strengths use and life satisfaction in under-graduate students (Douglass and Duffy, 2014), and vitality to be a mediator between strengths use and work performance (Dubreuil et al., 2014). Consistent with Froh et al. (2009), other factors could potentially impact the magnitude of not only strengths use on well-being, but also other relationships. As has been suggested elsewhere, these could include personality variables (cf. Sheldon and Lyubomirsky, 2004) or age (Froh et al., 2009). Also, given that strengths use may facilitate individuals feeling good about themselves, which in turn contributes to increased levels of self-esteem, it would not be surprising to find self-esteem moderating the relationship of strengths use to well-being. Moreover, how experienced a person is in drawing on and applying strengths may also influence the magnitude of the strengths use to well-being relationship. Thus, whether experience in applying strengths alters the strengths use to well-being relationship would represent a worthy question to address. According to these results, further investigations due to strengths use and related constructs need to consider mediator or moderator effects more carefully.

Additionally, given that strengths use is suggested to be more intrinsically motivated (e.g., Peterson and Seligman, 2004; Linley et al., 2010), the moderating role of motivation type (intrinsic vs. extrinsic) on the strengths use to well-being relationship could be explored, as too could the moderating role of motivation type on other potential mediators of the strengths use to well-being relationship; e.g., goal pursuit or need fulfillment (Linley et al., 2010). The extant body of self-determination theory research could prove insightful when exploring the relevance of motivations. In addition, given that the incidence of mental illness in society is increasing (Seligman et al., 2005), further attention needs to be given to the application of strengths use in respect to both ill-health prevention and positive health maintenance. Interventions promoting strengths use in education, work and private life may be a way to foster long-term individual resilience and optimal functioning with a favorable cost-value ratio [e.g., career counseling, study programs, further education, (social) media reports, articles, information from health professionals, etc.].

Finally, the implication of ‘having’ vs. ‘using’ strengths could be also focus of future research studies. Can one use an individual strength like one uses a bicycle? The language of ‘having’ vs. ‘using’ or ‘applying’ resembles to owning money (e.g., being rich) vs. spending money (e.g., investing, purchasing, donating, and the like). Personality characteristics can be seen as constructs, a summary of behaviors and they are not an existing entity. However, the questions is whether having a certain personality characteristic automatically implies using the associated behavior, or whether using the associated behavior (without having the associated personality characteristic) is also related to the outcome of interest? However, these questions ignore the environment and circumstances completely in which behavior of a person usually takes place (e.g., opportunity to demonstrate behavior due to situational factors).

## Conclusion

The results presented here make several useful contributions to the science how to potentially increase well-being of societies. First, the study confirmed the single-factor structure of the German version of the SUS and second, hypothesized relationships of strengths use and well-being indicators (positive inter-correlations with positive affect, self-esteem and vitality, negative inter-correlations with perceived stress and negative affect). Replications in social science research are of utmost importance to contribute to the establishment of evidence. Therefore, the German version of the SUS is psychometrically sound and the scale can be recommended for further research studies whether applying individual strengths can lead to, e.g., increased levels of well-being, health or any other outcome affected by strengths use. Future research should address for example, (1) if and how the promotion of applying individual strengths during education, can result in higher levels of well-being and healthiness in future lives, and (2) how the implementation of strength use in job-design guidelines or working conditions can contribute to higher levels of well-being.

The German version of the SUS was introduced and accordingly found to be well suited for studies with German-speaking adults. The revelation that using individual strengths is positively associated with well-being was reinforced. Clearly, there remains much to be done to scientifically explore the impact of individual strengths use in other countries and cultures.

## Availability of Data and Materials

The German version of the Strengths Use Scale (Fragebogen zur Anwendung von Stärken - FAS) can be found in the electronic Supplementary Material. All other personal data used for the statistical analyses are presented sufficiently within the paper.

## Ethics Statement

University of Innsbruck, vice director for research: Univ.-Prof. Dr. Sabine Schindler, Certificate of good standing, 62/2015. This document certifies that the Board for Ethical Questions in Science of the University of Innsbruck has reviewed the project ‘Well-being and Health of Medical Students and Practitioners’ of Dr. Thomas Höge-Raisig and Assoc.-Prof. Dr. Stefan Höfer. It is hereby certified that this project is in correspondence with all requirements of the ethical principles and the guidelines of good scientific practice of the University of Innsbruck.

## Author Contributions

The study was designed by AH and SH and carried out by them as well. Data were analyzed and interpreted by AH and DW. All authors contributed to the manuscript essentially (drafting, revising). All authors read and approved the final manuscript.

## Conflict of Interest Statement

The authors declare that the research was conducted in the absence of any commercial or financial relationships that could be construed as a potential conflict of interest.

## Abbreviations

CFA: confirmatory factor analysis
CFI: comparative fit index
df: degrees of freedom
EFA: exploratory factor analysis
FAS: Fragebogen zur Anwendung von Stärken
HI: higher bound
LO: lower bound
RMSEA: root mean square error of approximation
SE: standard error
SUS: Strengths Use Scale
SWB: subjective well-being
TLI: Tucker-Lewis-Index
VIA: values in action
VIA-IS: values in action-inventory of strengths
χ^2^: chi square-value
