# Supplementary material for: The German Version of the Strengths Use Scale: The Relation of Using Individual Strengths and Well-being
Source: Front Psychol. 2017 Apr 27;8:637. doi: 10.3389/fpsyg.2017.00637 (PMC5406780; doi:10.3389/fpsyg.2017.00637)
Supplement: Supplementary file 1 [file Presentation_1.pdf]

Fragebogen zur Anwendung von Stärken (FAS)

Die folgenden 14 Fragen beziehen sich auf Ihre Stärken, d.h. auf jene Dinge, die Sie **besonders gut** können. Bitte vergeben Sie jeweils eine Zahl zwischen **1** (trifft überhaupt nicht zu) und **7** (trifft genau zu).

		trifft überhaupt nicht zu →						trifft genau zu
		**1**	**2**	**3**	**4**	**5**	**6**	**7**
1.	Ich bin regelmäßig in der Lage das zu tun, was ich am besten kann.	☐	☐	☐	☐	☐	☐	☐
2.	Ich mache immer Dinge, die meinen Stärken entsprechen.	☐	☐	☐	☐	☐	☐	☐
3.	Ich versuche stets meine Stärken einzusetzen.	☐	☐	☐	☐	☐	☐	☐
4.	Durch den Einsatz meiner Stärken erreiche ich, was ich will.	☐	☐	☐	☐	☐	☐	☐
5.	Ich setze meine Stärken jeden Tag ein.	☐	☐	☐	☐	☐	☐	☐
6.	Ich setze meine Stärken ein, um das zu bekommen, was ich vom Leben erwarte.	☐	☐	☐	☐	☐	☐	☐
7.	Meine Arbeit bietet mir viele Möglichkeiten meine Stärken einzusetzen.	☐	☐	☐	☐	☐	☐	☐
8.	Mein Leben bietet mir die Möglichkeit, meine Stärken auf unterschiedliche Weise einzusetzen.	☐	☐	☐	☐	☐	☐	☐
9.	Meine Stärken einzusetzen ist für mich selbstverständlich.	☐	☐	☐	☐	☐	☐	☐
10.	Ich empfinde es als sehr einfach, meine Stärken einzusetzen.	☐	☐	☐	☐	☐	☐	☐
11.	Ich bin in der Lage, meine Stärken in vielen verschiedenen Situationen einzusetzen.	☐	☐	☐	☐	☐	☐	☐
12.	Die meiste Zeit verbringe ich mit Tätigkeiten, die ich gut kann.	☐	☐	☐	☐	☐	☐	☐
13.	Meine Stärken einzusetzen ist mir vertraut.	☐	☐	☐	☐	☐	☐	☐
14.	Ich bin in der Lage meine Stärken auf unterschiedliche Art und Weise einzusetzen.	☐	☐	☐	☐	☐	☐	☐

Huber, A., Webb, D., & Höfer, S. (2017). The German Version of the Strengths Use Scale: The Relation of Using Individual Strengths and Well-being. Frontiers in Psychology, 8:637. Supplemental material. doi: 10.3389/fpsyg.2017.00637
